# Underperformance of African Protected Area Networks and the Case for New Conservation Models: Insights from Zambia

**DOI:** 10.1371/journal.pone.0094109

**Published:** 2014-05-21

**Authors:** Peter A. Lindsey, Vincent R. Nyirenda, Jonathan I. Barnes, Matthew S. Becker, Rachel McRobb, Craig J. Tambling, W. Andrew Taylor, Frederick G. Watson, Michael t’Sas-Rolfes

**Affiliations:** 1 Panthera, New York, New York, United States of America; 2 Mammal Research Institute, Department of Zoology and Entomology, University of Pretoria, Pretoria, Gauteng, South Africa; 3 Zambia Wildlife Authority, Chilanga, Lusaka, Zambia; 4 Design & Development Services, Windhoek, Namibia; 5 Department of Ecology, Montana State University, Bozeman, Montana, United States of America; 6 Zambian Carnivore Programme, Mfuwe, Zambia; 7 South Luangwa Conservation Society, Mfuwe, Zambia; 8 Centre for African Conservation Ecology, Department of Zoology, Nelson Mandela Metropolitan University, Port Elizabeth, South Africa; 9 Centre for Veterinary Wildlife Studies, Faculty of Veterinary Science, University of Pretoria, Pretoria, South Africa; 10 Division of Science and Environmental Policy, California State University Monterey Bay, Seaside, California, United States of America; 11 Cape Town, South Africa; Università degli Studi di Napoli Federico II, Italy

## Abstract

Many African protected areas (PAs) are not functioning effectively. We reviewed the performance of Zambia’s PA network and provide insights into how their effectiveness might be improved. Zambia’s PAs are under-performing in ecological, economic and social terms. Reasons include: a) rapidly expanding human populations, poverty and open-access systems in Game Management Areas (GMAs) resulting in widespread bushmeat poaching and habitat encroachment; b) underfunding of the Zambia Wildlife Authority (ZAWA) resulting in inadequate law enforcement; c) reliance of ZAWA on extracting revenues from GMAs to cover operational costs which has prevented proper devolution of user-rights over wildlife to communities; d) on-going marginalization of communities from legal benefits from wildlife; e) under-development of the photo-tourism industry with the effect that earnings are limited to a fraction of the PA network; f) unfavourable terms and corruption which discourage good practice and adequate investment by hunting operators in GMAs; g) blurred responsibilities regarding anti-poaching in GMAs resulting in under-investment by all stakeholders. The combined effect of these challenges has been a major reduction in wildlife densities in most PAs and the loss of habitat in GMAs. Wildlife fares better in areas with investment from the private and/or NGO sector and where human settlement is absent. There is a need for: elevated government funding for ZAWA; greater international donor investment in protected area management; a shift in the role of ZAWA such that they focus primarily on national parks while facilitating the development of wildlife-based land uses by other stakeholders elsewhere; and new models for the functioning of GMAs based on joint-ventures between communities and the private and/or NGO sector. Such joint-ventures should provide defined communities with ownership of land, user-rights over wildlife and aim to attract long-term private/donor investment. These recommendations are relevant for many of the under-funded PAs occurring in other African countries.

## Introduction

Many African countries have designated generous proportions of their land surface as protected areas. Such protected areas vary greatly in their makeup, from strictly protected areas with no human settlement to areas that have resident communities where multiple uses of wildlife are permitted. African governments find it difficult to fund protected area networks adequately and are facing severe threats from poaching and human encroachment [1], [2]. These problems are pronounced where human settlement is permitted or tolerated inside protected areas, as occurs in parts of Ethiopia, Mozambique, Tanzania and Zambia, for example [3], [4].

**Figure 1:**
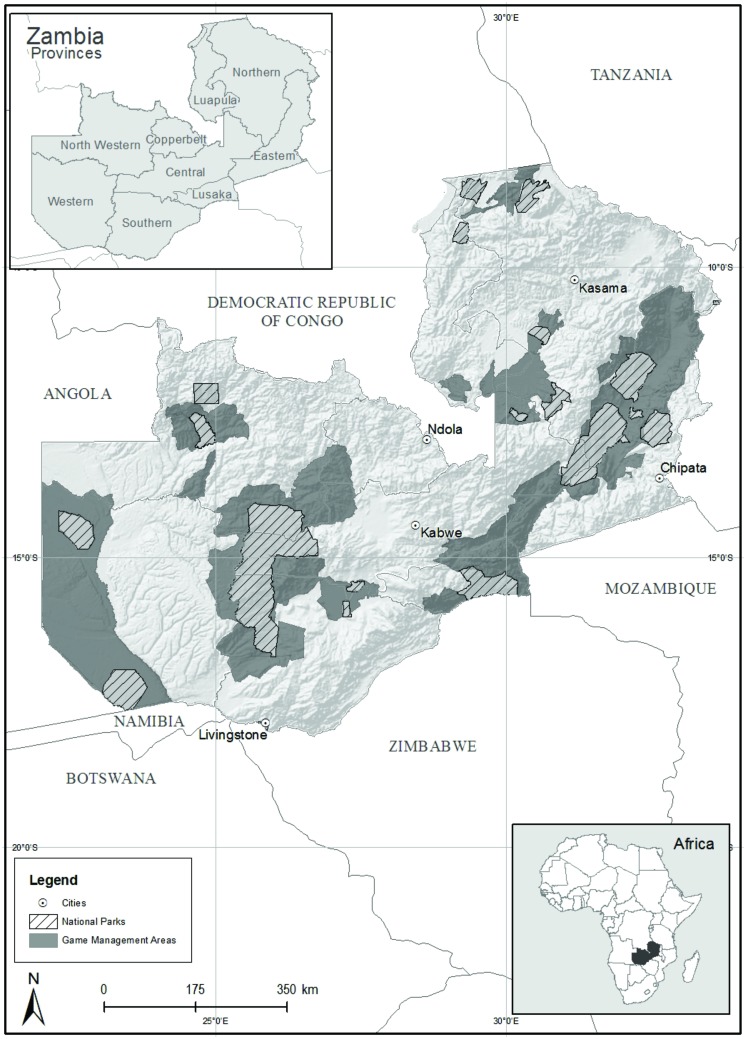
The Zambian protected area network.

Zambia has a vast wildlife estate encompassing 20 national parks (∼64,000 km^2^), 3 wildlife and bird sanctuaries (33.5 km^2^), 36 GMAs (167,000 km^2^) and several other protected area categories, comprising ∼40% of the nation’s land area [5] (Figure 1). Human settlement is not permitted in national parks, and land use is limited primarily to photo-tourism. National parks have generally not suffered from human encroachment, but are subject to widespread poaching, regular uncontrolled burning (which sometimes emanates from areas outside of the park boundaries) and in some cases, informal mining [6]. With the exception of Lusaka and Mosi-oa-tunya national parks, no protected areas in Zambia are fenced and most are simply demarcated with cut-lines or rivers, and in some cases, beacons. GMAs were established as buffer-zones for national parks and have been used primarily for trophy hunting in recent years [7]. Unlike in the national parks, settlement is permitted in GMAs and there are large and expanding human populations in many of them, which is accompanied by widespread habitat loss. Habitat destruction is exacerbated by shifting agriculture, charcoal production and in some cases, mining [7], [8], [9]. In both national parks and GMAs, wildlife is under severe pressure from poaching, both for bushmeat and for trophies such as ivory [10].

In the 1980s, there was recognition of a need for greater community participation in wildlife-based land uses in GMAs [8]. In the early 1980s, subsidiary legislation was introduced to partially decentralize authority over wildlife to communities [8]. The Zambia Wildlife Act of 1998 provided for establishment of ZAWA as a parastatal responsible for managing protected areas [9]. The Wildlife Act identified Community Resource Boards as the institutions for communities to co-manage and benefit from wildlife in GMAs [9] though no mechanisms were created to enable communities to benefit from wildlife in national parks.

Wildlife-based land uses in the PA network have potential to improve livelihoods significantly for communities. People in GMAs are poorer and less educated than the national average, and GMAs have low agricultural potential and offer few alternative livelihood opportunities [11]. Trophy hunting in the GMAs has potential to generate significant incomes for communities if wildlife populations are allowed to recover and systems are put in place to ensure equitable benefit sharing and best-practices [12]. Similarly, national parks have potential to benefit rural communities through tourism-related employment and business opportunities. The PA network as a whole has enormous potential to contribute to rural and national economic growth by providing the basis for development of a major tourism industry [13]. However, wildlife populations are waning in many GMAs and national parks, and incomes from both trophy hunting and photo-tourism are limited to fractions of the GMAs and national parks [7]. In addition, mechanisms to enable communities to benefit legally from the PA area network are limited. Consequently the PA network is under-performing in ecological, economic and social terms.

There have been several attempts by the Zambian government to address the underperformance of the protected area network. For example, in 2006, the Zambian Government embarked upon a reclassification programme for protected areas [5] and in early 2013, a moratorium was imposed on hunting in GMAs. In addition, two protected areas have been added to the estate in recent years: the ∼50 km^2^ Lusaka National Park and the 5,104 km^2^ Mukungule GMA. However, key challenges with regards to the functioning and effectiveness of Zambia’s PA network remain.

In this paper we provide evidence of the under-performance of the Zambian protected area network, give reasons for that performance and suggest interventions needed to make the system more effective in ecological, economic and social terms. These recommendations have relevance for the PA networks of many other African countries.

## Results and Discussion

### Ecological Indicators of Protected Area Performance

**Figure 2:**
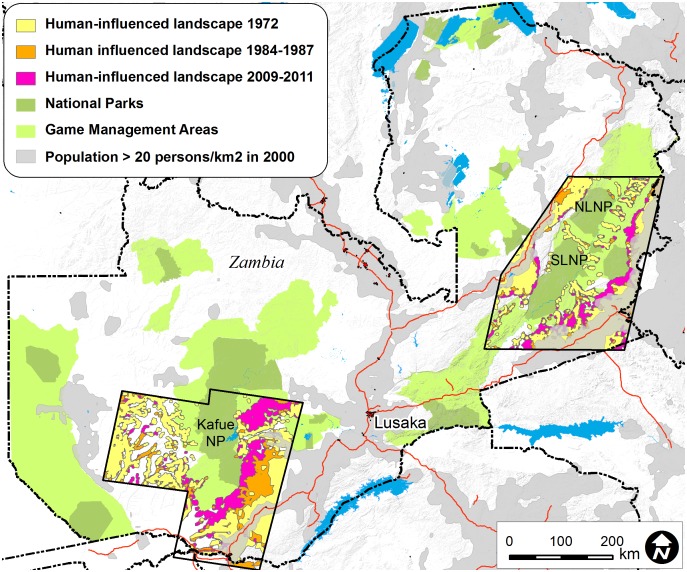
The extent of human encroachment of natural habitat in two focal areas in Zambia extracted from [6].

Human encroachment of protected areas in Zambia is worse than in most other African countries [14], and ∼2,500–3,000 km^2^ of land are deforested annually [15]. Human population growth rates in GMAs (2.49±0.18%) are higher than elsewhere (2.31±0.24%, T-test 0.577, d.f. = 70, p = 0.566) [16]. Almost 40% of the total area of GMAs is now comprised of human-modified habitat (c.f. 71.2% outside of the protected area network) [6]. By contrast, habitat loss in national parks is limited (2.1%) (Table 1). The rate of habitat loss in GMAs (0.69% conversion per year) is faster than in national parks (0.05%) or outside protected areas (0.51%) (Table 1, Figure 2, [6]). Extrapolating from [6]’s sample area (Table 1, Figure 2), ∼82 hectares of habitat are lost per daylight hour in GMAs on a national level. Human encroachment in GMAs is advancing from main roads towards national parks at a rate of up to 2 km per year ([6]). In some protected areas, and most notably Lukusuzi National Park [17], mining activity is evident and has potential to affect wildlife populations adversely through habitat degradation and bushmeat poaching [18]. In addition to habitat loss, human encroachment undermines the buffer zone role of GMAs for national parks, jeopardizes ecological connectivity among PAs and the concept of transfrontier conservation areas ([6]).

**Table 1:** Estimates of the extent and rate of habitat conversion in GMAs (from natural to human-modified habitat), national parks and land outside the protected area network in Zambia (taken from data extracted from [6]).

		∼1970		∼1985		∼2010		∼1970–∼1985		∼1980–∼2010	
Land type	Total in study area (km^2^)	Area human (km^2^)	% human	Area human (km^2^)	% human	Area human (km^2^)	% human	Increase per year (km^2^)	% increase per year	Increase per year (km^2^)	% increase per year
Whole study area	159805	58926	36.9%	60935	38.1%	80157	50.2%	134	0.08%	769	0.48%
NPs	27098	252	0.9%	257	1.0%	571	2.1%	0	0.00%	13	0.05%
GMAs	47430	9468	20.0%	10616	22.4%	18744	39.5%	77	0.16%	325	0.69%
Non-NP, Non-GMA	85277	49206	57.7%	50061	58.7%	60841	71.3%	57	0.07%	431	0.51%
Luangwa Valley GMAs	26502	6815	25.7%	6190	23.4%	8878	33.5%	−42	−0.16%	108	0.41%
Southern Kafue GMAs	20928	2652	12.7%	4427	21.2%	9866	47.1%	118	0.57%	218	1.04%

Data from aerial censuses indicate that wildlife populations in Zambian protected areas are relatively low (Table 2): ∼169,000 wild ungulates occur in the ∼61,000 km^2^ of Zambian national parks for which data are available (excluding species of the size of a bushbuck or smaller and hippos) and ∼143,000 in the ∼160,000 km^2^ of GMAs for which data were available. By contrast, ∼63,000 ungulates occur on <6,000 km^2^ of game ranches in Zambia [19]. Country-level population data for wildlife in other countries are scarce, but to provide a coarse comparisons, 1.8–2.8 million wild ungulates occur on 287,000 km^2^ of Namibian wildlife ranches ∼841,000 ungulates occurred on 27,000 km^2^ of Zimbabwean game ranches prior to the land seizures [20] and ∼215,000 ungulates occur in the ∼20,000 km^2^ Kruger National Park in South Africa [21], [22].

**Table 2:** Estimated wildlife populations in National Parks (data available for 61,462 km^2^ of the ∼64,000 km^2^), Game Management Areas (GMAs, data available for 159,654 km^2^ of the ∼167,000 km^2^) and game ranches (5,829 km^2^) in Zambia (excluding species of bushbuck size and smaller, and hippopotamuses for which count data were not available) (data taken from [19]).

Species	National parks	GMAs	Game ranches	Total
Lechwe	9,737	75,808	1,513	87,058
Impala	27,820	13,507	27,998	69,325
Wildebeest	47,815	4,069	630	52,514
Buffalo	21,301	15,938	2,107	39,346
Puku	16,838	7,529	4,904	29,271
Elephant	10,830	8,094	1,710	20,634
Sable	8,172	4,895	3,682	16,749
Zebra, plains	8,375	1,050	2,060	11,485
Waterbuck	5,254	2,333	2,987	10,574
Kudu	1,908	1,976	6,287	10,171
Hartebeest	4,429	3,952	2,051	10,432
Roan	2,384	1,632	1,647	5,663
Reedbuck	1,137	852	2,735	4,724
Eland	1,069	237	1,558	2,864
Tsessebe	990	88	410	1,488
Giraffe	579	178	321	1,078
Sitatunga	40	369	328	737
Nyala	0	0	95	95
	168,678	142,507	63,023	374,208

The biomass of large wild ungulates is lower in GMAs (mean 212±59 kg/km^2^) and national parks (791±240 kg/km^2^) than in extensive game ranches (2,424±305 kg/km^2^) (which are devoid of human settlement and rely primarily on trophy hunting for income) (Figure S1a) [19]. The diversity of wild ungulates is also lower in GMAs (4.7±0.58 species) and national parks (7.2±0.9 species) than on extensive unfenced game ranches 11.1±0.86 species) (Figure S1b) [19]. The higher biomass and diversity on private ranches is likely to be primarily due to the availability of greater resources for anti-poaching than in state protected areas. These findings reinforce the suggestion that trophy hunting need not have a negative impact on wildlife populations given appropriate land tenure arrangements [23].

Combining data from GMAs, national parks and extensive game ranches, wildlife ungulate biomass was negatively related to the presence of human settlement (in areas with settlement mean biomass was 268±70.8 kg/km^2^ c.f. 1,755±281 kg/km^2^), as was wild ungulate diversity (5.20±0.61 species c.f. 9.68±0.7) (F Ratio 26.2, d.f. = 2, p<0.001). Wild ungulate biomass was positively related to investment by the private sector/NGOs (in areas with such support, mean biomass was 1,592±222 kg/km^2^ c.f. 233±113 kg/km^2^ in areas without such investment, as was wild ungulate diversity (9.1±0.71 species c.f. 5.0±0.65) (F Ratio = 37.0, d.f. = 2, p<0.01) (Figures S2a, S2b).

**Figure 3:**
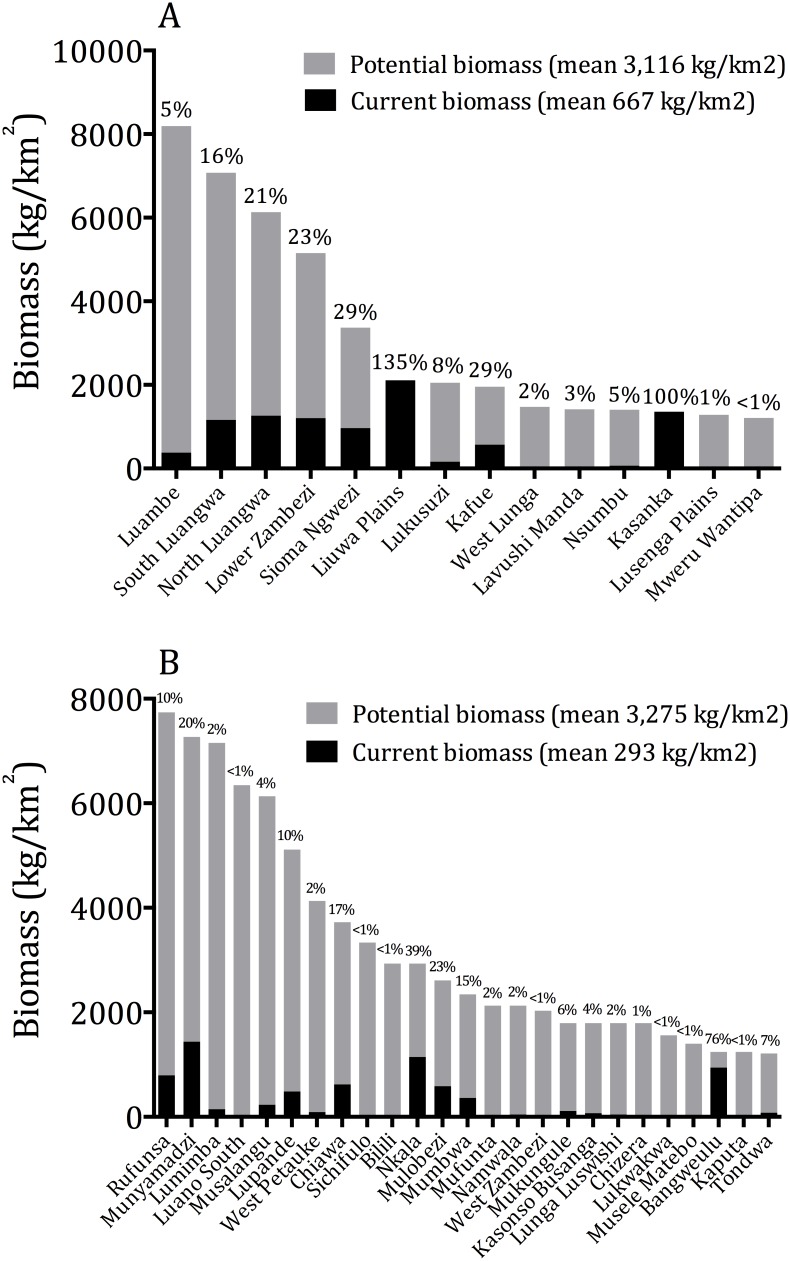
Observed large mammal biomass versus potential carrying capacity in Zambian (a) national parks and (b) GMAs.

Observed biomasses of large mammals in Zambian protected areas were lower than potential maximum carrying capacities by 93.7% in GMAs and 74.1% in national parks (Figures 3a, 3b). Wildlife densities are suppressed in some of Zambia’s ‘flagship’ national parks and GMAs. For example, biomasses in Kafue, South Luangwa and Lower Zambezi national parks are 29%, 16% and 23% of potential carrying capacity, whereas that in Lupande GMA (which has in the recent past been categorised as a ‘super-prime’ concession), stands at 11% of carrying capacity. Depressed prey populations means that predator populations are almost certainly also occurring well below historic densities.

Population trend data are generally not available for Zambian protected areas. An exception is the Luangwa ecosystem, where the biomass of wildlife declined significantly in all five GMAs and four national parks during 2011–2012, and 80% of species showed declining trends during 2009–2012 [24]. By contrast, in Liuwa Plains (co-managed by African Parks/ZAWA since 2003), wildlife populations have recovered and large mammal biomass (excluding hippos and species of bushbuck size and smaller) increased from 966 kg/km^2^ in 2003 to 1,921 kg/km^2^ in 2013 [25].

Declining wildlife populations in GMAs have been reflected in changes to the way ZAWA classifies GMAs. In 2008, 24 GMAs were classified as depleted or secondary whereas in 1997, only 16 were categorized as such [7].

### Economic Indicators of Park Performance

Zambia attracts fewer tourists than other African countries known for wildlife: South Africa (∼9.2 million), Botswana (∼2.3 million), Zimbabwe (∼2.2 million), Kenya (∼1.8 million), Mozambique (∼1.6 million), Tanzania (∼1.25 Million), Namibia (∼1.1 million), Zambia (∼0.9 million) (www.wttc.org, accessed July 2013). In 2005 (when the latest data on tourist visitation to parks were available), just 59,350 tourists visited Zambia’s national parks and 95% of those visited just five parks (Kafue, South Luangwa, Mosi-oa-Tunya, Lower Zambezi and Lochinvar): the remaining 15 parks comprising ∼32,000 km^2^ attracted just 1,384 tourists [26], [27]. Most national parks thus fail to attract enough tourists to support viable photo-tourism [27]. Earnings from national parks and GMAs in Zambia are low. Photo-tourism generated USD9.1 million for ZAWA in 2011 (USD142/km^2^) and UDD9.4 million in 2012 (USD146/km^2^) (ZAWA unpublished data). The majority of those earnings (96.9%, USD154/km^2^) were from four parks (South Luangwa – 44.4%, Mosi-o-tunya – 23.7%, Kafue – 14.7% and Lower Zambezi – 14.1%), which comprise 36,681 km^2^ (57.3%) of the national parks network (ZAWA unpublished data). The remainder of the parks generated just USD182,229 (USD6.7/km^2^) due to the relative (or actual) absence of tourism operators. Photo-tourism operations exist in only 10 of 36 GMAs [7] and in a marginal capacity given they currently are unable to bid for concessions in GMAs. Photo-tourism operations in GMAs are generally limited to lodges on national park boundaries with little investment or activity in the GMAs.

From trophy hunting income, ZAWA only earned concession fees from ∼51% of the GMA estate in 2012 (ZAWA unpublished data). ZAWA earned a total of USD4.34 million from trophy hunting and resident hunting respectively in 2012 (equating to USD26/km^2^) (ZAWA unpublished data). We estimate that an additional USD16.1 million was earned by operators from trophy hunting in GMAs, yielding total earnings of USD97/km^2^ (averaged across all GMAs including those not hunted, Table 3). Earnings per km^2^ from trophy hunting (when excluding hunting areas that do not generate any income to allow for comparison with other countries) (US$291±116/km^2^) are lower than most other SADC countries: Zimbabwe – USD1,028/km^2^; Tanzania – USD424/km^2^; Namibia – USD378/km^2^; Mozambique – USD130/km^2^, [28] (F-Ratio 11.0, *d.f*. = 1, p = 0.001). Within Zambia, gross earnings per km^2^ from trophy hunting in GMAs (USD97/km^2^) are markedly lower than on extensive game ranches (USD878±226) (F Ratio 15.9, d.f. = 1, p<0.001) [19]. Contributing to the low earnings from hunting in Zambia in 2012 was the fact that 19 leases for hunting concessions ended prior to the end of the hunting season, and resident hunting was halted by the Zambian government (ZAWA unpublished data).

**Table 3:** Gross earnings in USD from trophy hunting and non-resident hunting in Game Management Areas in 2012 (excluding land under 99 year lease within or near to GMAs) (NB that operators’ net earnings are markedly lower than the gross income, due to the costs associated with paying concession and animal license fees to ZAWA, the costs of running and marketing safaris, and the costs of managing the concessions).

	Total	Operators*	ZAWA*	Community Resource Boards*	Chiefs*
Trophy hunting					
Concession fees	N/A	N/A	580,572	108,857	36,286
Animal license fees	N/A	N/A	1,697,878	1,528,090	169,788
Operators license fees	N/A	N/A	80,500	0	0
PH license fees	N/A	N/A	65,621	0	0
Daily rates	N/A	11,655,430	N/A	N/A	N/A
Trophy fees	N/A	4,506,987	N/A	N/A	N/A
Sub total	16,162,417	16,162,417	2,424,571	1,636,947	206,074
Resident hunting					
Total earnings	88,932	N/A	44,489	40,040	4,449
Non resident hunting earnings/km^2^	0.6	N/A	0.3	0.3	0.0
Total earnings	16,251,349	16,162,417	2,469,060	1,676,988	210,522
Total earnings/km^2^	97	N/A	14.8	10.0	1.3

*NB Operators were assumed to generate the total gross income from trophy hunting, from which the ZAWA and community income is derived. This income excludes that generated from extensive game ranches, which are on 99 year lease.

### Social Indicators

The primary benefit to communities from national parks is employment and the development of wildlife-based economies around tourism hubs such as the growth point of Mfuwe, adjacent to South Luangwa National Park. Tourism generates an estimated 19,000 jobs in Zambia [27], though the proportion derived specifically from parks is not clear. In Mfuwe, ∼900 jobs are created and such workers receive mean salaries of US$450/month, ∼3x the minimum wage (A. Coley, pers. comm.). Tourism-related employment is significant because it can result in improved attitudes towards wildlife conservation [29]. However, most parks generate virtually no employment for communities due to the lack or small-scale of tourism operations.

In GMAs, benefits accruing to communities from trophy hunting include income generation for Community Resource Boards, employment and in some cases, various forms of development assistance from hunting operators. In some GMAs, such as those in the Luangwa Valley and Bangweulu system, livelihood improvements associated with income from trophy hunting are significant [30]: families in the most wildlife-rich GMAs are ∼17% better off than those outside of the GMAs, and have a 7.8% higher chance of obtaining employment [8]. However, no earnings whatsoever are generated in half of the GMAs, and average earnings accruing to communities across all GMAs are low (USD11.9/km^2^) (ZAWA unpublished data, Table 3). Concurrently, communities incur significant costs as a result of living with wildlife and ∼50 people are killed annually by wild animals [31]. Overall, communities living in GMAs are 30% poorer than the national rural average [32].

### Reasons for the Under-performance of Protected Areas

#### 1. Community-related issues

There are no legal mechanisms to enable communities to benefit financially from photo-tourism in PAs. Furthermore, photo-tourism is under-developed and is practiced in a small fraction of the PA estate where the resource and existing conditions are suitable. For example, photo-tourism operations exist in only 10 of the 36 GMAs [7] and the existing legal framework does not require photo-tourism operators to pay Community Resource Boards [9]. Consequently, communities are only able to derive legal benefits from wildlife via trophy hunting in GMAs and through employment in national parks and GMAs.

The Wildlife Act fails to recognize communities as the rightful owners of the land or wildlife in GMAs (in contradiction to the Lands Act of 1995, [33]). There are no mechanisms for specific communities to obtain exclusive rights over land in GMAs or over the wildlife resources therein. Consequently, mechanisms to prevent in-migration of external communities into GMAs are weak. Immigration is likely fuelled by the availability of bushmeat, firewood and other natural resources, and in some cases, due to potential employment opportunities from tourism in adjacent national parks. Clearing of lands for settlement and shifting agriculture and tree-cutting for charcoal production all contribute to habitat loss in GMAs [9], [15]. Uncontrolled immigration means that land use planning is difficult to enforce [6] and creates an open-access system whereby it pays communities to occupy land or kill wildlife before someone else does.

ZAWA collects revenues from trophy hunting and remits 20% of the concession fees to communities and 50% of animal license fees for wildlife shot as trophies in GMA [8]. ZAWA thus retains most of the income from hunting in GMAs even though the land is under customary tenure and belongs to the community. Wildlife-based land uses are effectively heavily taxed, whereas livestock production, small-scale agriculture and others are not (Table 3). Furthermore, income from wildlife is often paid late and as hand-outs that do not create a clear link between conservation and earnings [9], [30]. Earnings for communities from trophy hunting are lower than estimated earnings from illegal bushmeat hunting and create weak incentives for conservation [34].

Communities living in GMAs are also marginalized from the decision-making relating to wildlife management. The concession agreements that are signed between ZAWA, hunting operators and Community Resource Boards on the leasing of GMAs favour ZAWA. ZAWA retain most of the income, delegate numerous responsibilities to other stakeholders, allocate minimal rights, are able to dismiss Community Resource Boards or to cancel the leases of hunting operators, and are not obliged to make their financial records available [11]. There is widespread resentment towards ZAWA, hunting and tourism operators (anon survey respondent, pers. comm.).

The make-up and functioning of Community Resource Boards is also problematic. More affluent community members benefit disproportionately from trophy hunting [30]. There is a lack of appropriately educated or skilled community members to form Community Resource Boards, which limits their ability to negotiate effectively with ZAWA or operators [9]. Skills shortages are exacerbated by the fact that the Community Resource Boards are re-formed each 2–3 years [35]. Furthermore, local chiefs can dismiss board members and ‘village scouts’ (community members employed by the community resource boards for anti-poaching) at their discretion, making Community Resource Boards unstable. There are frequent financial irregularities associated with CRB income from wildlife and funding allocated to resource protection is inadequate [9]. The 850 village scouts employed throughout Zambia are poorly and irregularly paid, insufficiently trained or equipped and are too few in number to effectively patrol the vast GMA estate [11].

#### 2. ZAWA-related issues

When ZAWA was formed in 2000, a key objective was to increase efficiency and financial self-sufficiency [35]. ZAWA was meant to retain government support, but in practise funding was reduced to just 15% of operational budget. Including the ∼USD4.6 million generated from trophy hunting, ZAWA’s resources equate to USD20–60/km^2^/year, which compares poorly with the USD358–USD455/km^2^ required to manage protected areas effectively [36] ZAWA has a field staff complement of 1,179 to protect 231,000 km^2^, which equates to 1/196 km^2^ and compares poorly with the southern African regional average of 1/40 km^2^ [1]. As little as 8% of the ZAWA budget is spent on GMAs even though they generate >50% of ZAWA earnings and comprise >70% of land under their jurisdiction [7]. Furthermore, ZAWA is encumbered by large numbers of sick and poorly trained staff, and an increasing proportion of ZAWA funds have been accruing to head office (anonymous survey respondent pers. comm.). Consequently, field capacity is low when the threat to wildlife from the bushmeat trade and is unprecedented and that from ivory poachers resurgent [2], [37]. Funding shortages mean that ZAWA’s mandate of protecting the vast wildlife estate is impossible to achieve.

To rectify this situation, ZAWA have allocated partial responsibility for resource protection to Community Resource Boards and hunting operators with the effect that roles are blurred and none of the stakeholders contribute sufficiently. This is despite the fact that responsibility for anti-poaching falls on ZAWA according to the Wildlife Act [11]. Technical support to Community Resource Boards from ZAWA is inadequate and communication between Community Resource Boards and ZAWA is limited [9].

Forced to generate their own funding, ZAWA rely on safari hunting in GMAs for ∼45–67% of their revenue [11], [35]. This reliance means that ZAWA are sometimes forced to make decisions to achieve financial survival at the expense of the wildlife they are mandated to conserve. For example, in 2003, ZAWA increased quotas and reduced the size of hunting blocks [7]. In addition, ZAWA have imposed high ‘fixed-quotas’ (of 60–100%) whereby operators are forced to pay for animal license fees before commencement of hunting [7], [11]. Such quotas create a perverse incentive, forcing operators to harvest wildlife regardless of sustainability. Due to lack of funds, there is a lack of monitoring of wildlife populations or of trophies. Trophy quotas are established arbitrarily, quota utilization is low (averaging 40%) and prior to the ban trophy quality was falling, implying that quotas were not sustainable [7]. Quotas of lions have been particularly excessive [38], [39].

The requirement for ZAWA to generate their funds means that it is not in their best interests to devolve user-rights over wildlife to communities in GMAs or to private landowners on extensive wildlife ranches (who are forced to pay license fees for animals hunted to ZAWA), as that would reduce income in the short-term and create perceived competition [19]. A similar conflict arose when the Zimbabwe Parks and Wildlife Management Authority became a parastatal responsible for generating their own revenue [40]. In Zimbabwe, that shift resulted in a gradual shift towards centralized authority over wildlife and gradual reversal of the devolution that made the wildlife ranching industry in that country such a success [40].

Wildlife in GMAs is affected by several other forms of legal harvest including resident hunting conducted by Zambian citizens and residents. Resident hunting licenses cost ∼1/3 the meat value of the animals hunted, and consequently resident hunters often shoot wildlife specifically to obtain meat to sell, and in many cases, quotas are exceeded (Table 4). Such abuses are made possible by inadequate supervision of resident hunts and corruption. Prior to the hunting ban, a varying amount of wildlife was also killed under ‘special licenses’ allocated on a discretionary basis by the Minister of Tourism and Arts [35]. Special licenses and non-resident hunting licenses strip the value of wildlife and create minimal incentives for conservation by communities. The multiple forms of legal off-take compound the effects of habitat loss, predation and poaching and confer heavy depletion of wildlife in most GMAs (Figure 3b).

**Table 4:** The price of citizen licenses for hunting in GMAs, the meat value of those species and the value of the meat relative to the price of citizen licenses.

	Citizens	Meat value*	Meat value relative to license fee
Buffalo	493	1398	3
Bushbuck	40	142	4
Bush pig	16	146	9
Duiker	32	40	1
Eland	592	1425	2
Hartebeest	158	355	2
Impala	40	150	4
Kudu	493	551	1
Oribi	59	33	1
Puku	69	159	2
Reedbuck	79	159	2
Warthog	79	185	2
Waterbuck	158	615	4
Wildebeest	158	593	4
Zebra	296	757	3
Average ± S.E.			3±0.75

*Assuming male animals are hunted, a meat price of USD4.3/kg (the price paid by Lusaka butchers for dressed meat) and assuming the mean mass of dressed carcasses presented by [72].

#### 3. Operator-related issues

In national parks, tourism operators are not required to conduct anti-poaching and input is generally limited to sporadic provision of support for NGOs involved in resource protection [41]). In GMAs, the concession allocation system created disincentives for investment and good practice by hunting operators. Leases are granted for 10 years (or 15 for depleted blocks), which is not sufficient to encourage adequate investment in the area or to a sense of ownership of the areas [42]. Where wildlife populations are depressed, 15 years does not allow for sufficient time for operators to recoup the investments needed to allow wildlife populations to recover [43].

The hunting concession agreements (which outline commitments to anti-poaching and community outreach) are not effectively enforced [9]. Furthermore, operators typically vacate the hunting blocks during the rainy season, leaving their areas vulnerable to poachers. Some of the hunting blocks in GMAs are extremely large, and several operators complained that their size renders effective enforcement impossible. Some operators appear to invest a significant amount in anti-poaching and others virtually nothing. However, there is no system to link past performance of hunting operators to the prospects of them obtaining an extension of a lease or a new area. Consequently, responsible operators are not adequately rewarded, and unscrupulous operators not adequately punished, reducing incentives for good practice and allowing abuses (including alleged over-shooting of quotas by some operators) to continue [44]. In general, declining wildlife populations have resulted in falling incomes [7] and thus declining resources available protect the resource.

In early 2013, ZAWA imposed a moratorium on hunting in GMAs in response to alleged corruption in the tender process and due to concern over wildlife population trends. Consequently, hunting operators have vacated the GMAs, resulting in loss of their contribution to anti-poaching and creating a vacuum in which illegal activities are more likely to proceed unhindered. Evidence from Kafue National Park suggests that the simple presence of operators has a significant deterrent effect for poachers [41]. Furthermore, there have been extended periods in 2013 when village scouts went without pay, and many likely relied on poaching for income (Anon survey respondent, pers. comm.). While government has subsequently stepped in to pay the salaries of village scouts, it is not clear as to how such payments will be sustained in the absence of hunting income. There have been few proposals from the photo-tourism industry to take over GMAs in the wake of the hunting ban. The hunting moratorium is thus likely to fuel wildlife declines by reducing: anti-poaching effort and presence in GMAs; working capital for ZAWA; and incentives for conservation by communities.

#### 4. Other factors

There are multiple authorities in GMAs with jurisdiction over the management of different resources [35]. In addition, conflicting legislation precludes effective land use planning. For example, the wildlife act states that ZAWA is responsible for wildlife resources in the area, and by implication habitat, whereas the Local Government Act says that local councils are responsible for planning and development [35]. Consequently development-related decisions are sometimes made in GMAs with little consideration of their impacts on wildlife or the potential for wildlife-based land uses. Furthermore, chiefs are able to allocate land to private investors in the middle of GMAs without consulting Community Resource Boards [9].

### Changes Needed to Improve the Functioning of the Protected Area Network

In this section we outline a number of key steps that we consider to be necessary to improve the functioning of the Zambian PA network, many of which are likely to apply to PAs in various other parts of Africa.

#### 1. Empowering communities to benefit more from the PA network

There is a need to develop systems to enable communities to benefit more from PAs so as to enhance their social and economic value. Potential models to for such community engagement are discussed in the next section.

#### 2. Re-defining the role of ZAWA

The role of ZAWA should be re-defined so that the organisation focuses primarily on national parks, and elsewhere plays more of a hands-off coordinating role to facilitate the development of wildlife-based land uses by community, private, and NGO partners. There is need for recognition from government that unless earnings are vastly higher than they have been in the past, the GMAs cannot be used to fund other parts of the PA network or to support ZAWA headquarters. If ZAWA continue to extract revenues from GMAs, then a commensurate reinvestment in law enforcement and wildlife management in those areas is required.

#### 3. Greatly increasing funding from government for ZAWA

Drastically increasing government funding for ZAWA. Such funding should be seen as an investment in the development of the tourism industry rather than as a cost (as discussed further below). Even at current rates of funding for ZAWA, there is evidence of impressive returns on investment: in 2005, the government of Zambia received USD8 million from wildlife-related tourism from an investment of just USD1 million [32]. The tourism industry in Zambia comprises just 5% of GDP compared to a global mean of 14%, and creates just 3.6% of national employment compared to a regional mean of 7.2% [13], [45], [46]. In addition, there has been relatively minimal capital investment in the tourism industry in Zambia (1.7% of GDP c.f. a regional average of 6.9%) [13]. There is thus major scope for growth in the Zambian tourism industry, particularly given projections of rapid increases in visitor arrivals to the SADC region [13]. To achieve that growth, however, there is a need for much greater investment in protecting the wildlife product.

#### 4. Harnessing international willingness to pay for conservation

There is a need for greater international support for PA management in Zambia. The Zambian PA network constitutes ∼40% of the country’s land area, compared to a global average of 12% [47]. A developing country such as Zambia cannot reasonably be expected to pay for all of the costs of the maintenance of that global asset in addition to bearing the associated opportunity costs. Furthermore, tourism and trophy hunting (in the case of GMAs) may never generate enough income to cover the costs needed for effective protected area management. There are numerous potential avenues for harnessing international willingness to pay for wildlife conservation, including *inter alia*:

*a) Encouraging co-management agreements for PA management.* In national parks, ZAWA could pursue the development of co-management agreements with NGOs and the private sector to share the burden of PA management and attract additional technical capacity. Encouragingly, there has been an increasing trend towards development of such agreements in Zambia in recent years [5], [48]. There are official co-management agreements in place for five national parks and two GMAs (ZAWA unpublished data). In addition, there is significant NGO involvement in resource protection in several other national parks (e.g. Kafue, South Luangwa, Lower Zambezi, Sioma Ngwezi, and Nyika) and 11 other GMAs. If ZAWA could focus primarily on coordinating and regulating the management of PAs by partner organisations, the effectiveness and cost-effectiveness of parks could be greatly enhanced. NGOs and the private sector could be encouraged to take over the management of entire PAs or of concessions within PAs. Such a set up also would provide NGOs that are opposed to trophy hunting with the opportunity to reduce the prevalence of the practice by making payments in lieu of hunting revenues in the GMAs.

*b) Attracting funding for the development of a national CBNRM programme.* In addition to funding ZAWA and the PA network, there is a need for funding to allow for a national community-based natural resource management (CBNRM) programme to facilitate the capture of a greater proportion of benefits from the PA network by communities. Such funding could build on the progress made by the Administrative Management Design for Game Management Areas (ADMADE) during the 1990s. The successes of the Namibian community conservancy programme and the Zimbabwean CAMPFIRE programme have been dependent on long-term and substantial injections of technical capacity and funding (USD173 million and USD35 million respectively) ([49], C. Weaver pers. comm.). Likewise, the community conservancies in the northern Kenyan rangelands are supported by a coordinating NGO with funding of ∼USD1.2 million annually [50]. A similarly well funded, supported and coordinated national CBNRM programme is needed in Zambia.

*c) Encouraging allocation of a greater portion of overseas development aid towards PAs.* There is a strong case for donors to direct a portion of international development aid towards PA management, capitalizing ZAWA and/or co-management agreements between ZAWA and NGOs, and/or developing a national CBNRM programme. Recent estimates suggest that for every 1% increase in tourism-related investment in the SADC region, a 0.3% increase in GDP per capita accrues [13]. An allocation of just 2–3% of the ∼USD1 billion of overseas development aid that Zambia receives annually [51] would cover the costs of managing the national parks effectively and of protecting the main tourism asset [32]. However, there is a need for checks and balances to ensure efficient use of donor funds by ZAWA.

A potentially cost-effective way of using donor funds to achieve both conservation and development objectives (ideally in the context of a coordinated national CBNRM programme) is through schemes that channel payments to communities living in or near PAs for the provision of ecosystem services. For example, communities could be paid an annual fee for desisting from converting habitat or for protecting wildlife from poaching. In the Maasai steppe in Tanzania for example, such an approach has achieved notable conservation gains for a cost of just USD48/km^2^ [52], which compares favourably with the costs of traditional PA management. A key potential value of PES approaches is that they can help correct ‘market failures’ whereby wildlife that is valuable to the nation as a whole is not valuable to the people living with it, who thus over-exploit the resource or invest little in protecting it [50]. PES approaches could be combined with efforts to provide communities with stable markets and fair prices for livestock and crops, as is being conducted in the Kenyan community conservancies, and as part of the community markets for conservation approach in parts of Zambia [50], [53]. However, the latter approaches are only likely to be successful if combined with efforts to actively protect wildlife populations via anti-poaching.

*d) Other options.* Capturing the willingness of international philanthropists to pay for conservation represents another potential means of funding PAs [54]. Several precedents for such investment exist, such as that provided in Gorongosa National Park in Mozambique and the Grumeti Game Reserve in Tanzania. Additionally, attracting carbon-related investment via projects such as REDD+ could potentially generate funds for the protection of woodlands and associated biodiversity, notwithstanding the constraints currently associated with that programme [55]. Further, government could potentially generate additional revenue by taxing stakeholders who benefit substantively from ecological services provided by PAs, such as commercial farmers and power-generating or mining companies.

#### 5. Attracting significant private investment

To develop tourism and hunting businesses in national parks and GMAs, and to protect and manage wildlife in GMAs (assuming that ZAWA focuses their efforts on national parks) would require substantial private and/or donor investment. Rehabilitating a single depleted GMA is predicted to cost millions of dollars and such investments would likely take many years to recoup [43]. Attracting such investment is most likely under the following circumstances: an enabling policy environment; simple safe and standardized processes for investing; long leases (of at least 40 years for depleted areas [43]); attractive terms (e.g. as would be conferred if ZAWA desisted from taxing wildlife-based land uses in GMAs); minimal red-tape or interference from ZAWA, a functioning national programme for the development of community wildlife conservancies (see below) and knowledge that communities are supportive of wildlife investments on their land. In GMAs, investors and community partners should be able to choose any forms of wildlife-based land uses and the combinations that will yield the best returns for their particular spatial setting. Consequently, hunting bans or bans on the hunting of high-value species are to be avoided so long as hunting can be managed in a manner that ensures sustainability [12]. Finally, resident hunting should not be allowed to undermine private investments in GMAs and should only be permitted if desired by the community and investor partners and if priced appropriately.

#### 6. Addressing key conservation threats decisively

For the PA networks to function better, there is a need to decisively address key threats such as human encroachment, bushmeat and other forms of poaching. Human encroachment could be addressed through linking the allocation of leases to communities with agreed land use plans (see below). Alternatively, portions of GMAs close to national parks could be re-gazetted as ‘buffer-zones’ where human settlement is not permitted (though the leases for such areas could still be leased to communities to enable them to benefit from legal wildlife-based land uses, see below). In such instances and at the edge of community conservancies (see below), fencing external boundaries (if supported by communities) may play a significant role in reducing edge-effects, reducing human-wildlife conflict, demarcating boundaries and helping to prevent further encroachment [58], [59]. Where human settlement has reached right up to the boundaries of national parks, the fencing of such sections may be justified if appropriate materials are used and if adequate funding for maintenance exists.

Addressing poaching requires elevated opportunities for communities to benefit legally from wildlife, stiffer legal frameworks relating to poaching (with penalties that reflect the value of wildlife and the threat posed by poachers to the life of PA staff) and improved anti-poaching are required [2]. To achieve professional, well-funded anti-poaching requires either a much greater investment from ZAWA, and/or significant investments from the private and/or NGO sector.

#### 7. Prioritising conservation efforts

Zambia’s PA network is vast and funding limited. Furthermore, some of the GMAs are probably damaged beyond repair due to heavy human settlement and habitat modification. Given these factors plus the high human population growth rates, conserving the entire PA network in the long term is unlikely. There is a case for a scientific priority setting exercise to identify the PAs that should be the priority for investment of available funding.

### Potential Models for Achieving Elevated Community Participation in the PA Network

The degree of involvement of communities in national parks versus GMAs should arguably differ as people reside the latter but generally not the former. For national parks, one option would be to allocate ownership of PAs, shareholdings of PAs, or tourism concessions within parks to neighbouring communities for them to lease out to tourism operators. Precedents for such arrangements have been established in South Africa, for example, through the creation of contractual parks [56]. Such changes would require clear definition on who comprises ‘the community’ as at present, membership is poorly defined, compromising effective plan implementations and revenue sharing.

In the GMAs, we recommend changes that empower communities and enable them to participate in and benefit from wildlife-based land uses through the formation of Community Wildlife Conservancies (CWCs). Precedents for such conservancies have been developed in both Namibia and the northern rangelands of Kenya, both of which have achieved significant conservation and livelihood gains [50], [57]. While there are many potential variants of such models that could applied to the GMAs, there are a few general principles that we believe should be adhered to: a) communities should be allocated the maximum permissible degree of ownership over land and wildlife; b) that ownership should be structured such that it is exclusive for specific communities to avoid perpetuation of the tragedy of the commons; c) communities should accrue benefits from wildlife directly, and not via remittances from ZAWA; d) communities must actively participate in wildlife management decisions and not be passive recipients of hand-outs; e) community structures that are used to administer finances relating to wildlife must be democratic, transparent and regularly audited to ensure equitable distribution of benefits and avoid elite capture, and f) there must be mechanisms to ensure funding for high-quality anti-poaching security given the level of threat in GMAs. Two examples of potential models for the establishment and functioning of CWCs in GMAs are as follows:

#### 1. Complete devolution model

Here CWCs in the GMAs would be established as community conservancies based on joint ventures between communities and the private sector. ZAWA would not extract income from the CWC and would play a purely regulatory, facilitating and over-seeing role. ZAWA currently earn nothing from half of the GMAs as it is and so in such areas this kind of arrangement would not cause loss of revenue for ZAWA. The community would create a democratic, accountable and transparent body or trust to administer the area as a conservancy. A long lease would then be allocated by government to that community conservancy trust, the validity of which should be contingent on a land use plan that ensures that a particular area is set aside for wildlife only. Communities would then sub-lease the land to or engage in business partnerships with private or NGO investors, ideally for long periods to attract significant investment. That leasing process could follow either a public auction or an open tender process. The communities and successful bidding investors would then form a second body or trust with the mandate of managing wildlife in the GMA, ensuring professional anti-poaching and effective communication and cooperation between the community and investors. Alternatively, the investors could gain representation on the community conservancy trust after signing a partnership agreement, and then that body would coordinate wildlife management. Investors would then pay: a) an annual land rental to communities (which means they would derive some income without waiting years for wildlife populations to recover); b) an annual resource use fee (e.g. bed night levies or licence fees for animals hunted) (which means that communities would receive income proportional to their conservation ‘performance’); and, c) an annual levy to capitalize the body with the responsibility for managing wildlife. Investors could generate income either by acting as their own hunting or tourism operators, by auctioning hunting packages to the highest bidding operator, or sub-leasing tourism concessions.

#### 2. Partial devolution model

In this model, GMAs would be administered as community conservancies based on tripartite public-private-community-partnerships involving communities, ZAWA and private investors. As in the previous model, the communities would form a body that obtains a lease for the land, and would sub-lease the land to investors (or engage in a long term business partnership). A largely independent not-for-profit body would be established with representation from ZAWA, investors/participating NGOs and communities with the mandate of managing the wildlife in the area. The fees that have traditionally been paid to ZAWA by operators would be paid into that not-for-profit body to ensure that they are reinvested in the area.

Re-designating GMAs as CWCs would confer multiple benefits: CWCs would secure land rights for communities and protect against the loss of land and natural resources that would arise from the current open access system; CWCs could provide an effective buffer role for national parks; communities would generate significant and sustainable incomes, meat supplies and employment; if wildlife populations were successfully rehabilitated, CWCs could generate economic outputs at least 20x greater than currently being earned in GMAs [43]; CWCs would attract external investment by a wide-spectrum of donors; CWCs would create scope for communities to sell carbon and biodiversity credits by securing land rights [15]; and ZAWA would be relieved of the burden and costs of protecting wildlife in GMAs.

### Legislative Changes Needed to Allow CWCs to Happen

Ownership of wildlife in Zambia is vested in the President on behalf of the country [19]. On private land, user-rights over wildlife can be conferred to landowners via certificates of ownership, but there is no such provision for communities. Similarly, there is scope for investors, but not communities, to alienate land [9]. New legislation is required to enable communities to obtain 99-year leases for their land in GMAs following formation of a CWC, and to enable them to obtain full-user rights over wildlife.

At present, fencing is a pre-requisite for obtaining ownership over wildlife on wildlife ranches in Zambia [19]. In some contexts, as discussed, fencing can confer clear benefits [58], [59]. However, fencing can also reduce ecological connectivity and provide massive supplies of snare-material if inappropriate wires are used [58], [60]. Fencing should not be a pre-requisite for communities to obtain user-rights over wildlife and should not be permitted between adjacent CWCs or between CWCs and national parks. If needed, fencing should also be composed of kinked wire mesh to prevent snare construction, and should be accompanied by an environmental impact assessment and a clear long-term maintenance plan and budget.

### Conclusions

Wildlife populations are faring poorly in many African protected areas [61] and many of the challenges and solutions highlighted in this paper occur in other African countries. The under-funding of protected area networks is a widespread problem and parks agencies are often required to generate their own income, which creates the kinds of conflicts of interest outlined in this paper. There is a need for vastly elevated funding for PA management from both African and international governments and institutions. There is the need for improved mechanisms to enable communities to participate in and benefit more from wildlife in many African countries. In addition, creating frameworks for safe and secure private and NGO investment in PA management is an intervention with widespread applicability. Strong measures to address unplanned human encroachment in PA networks are also needed in many areas, as are efforts to tackle high levels of ivory and bushmeat poaching. The net result of these interventions is likely to be significant improvements in the effectiveness of parks networks, substantial job creation and economic gains due to growth in tourism industries. In the absence of such changes, wildlife populations in protected areas in Zambia and many other countries are likely to continue to wane due to on-going poaching and human encroachment.

## Methods

### Insights into the Performance of Protected Areas

We provided insights into the performance of PAs using: a) a literature review; b) data obtained from ZAWA and other sources; c) and, semi-structured interviews with key stakeholders, including the highest-ranking ZAWA officials (n = 7); representatives from relevant NGOs (n = 14); wildlife industry experts/photo-tourism operators (n = 11); and trophy hunting operators (n = 13). The literature review was conducted using key words such as ‘Zambia’, ‘GMAs’, ‘wildlife policy’, ‘CBNRM’, ‘ADMADE’, ‘trophy hunting’, ‘wildlife ranching’, ‘co-management’, ‘bushmeat’, ‘and encroachment’, etc. We included both published papers and unpublished consultancy reports. We searched for references using Google and Google Scholar. Selection of survey respondents was conducted by contacting and meeting as many individuals from each group that we could during our fieldwork period (September–November, 2012). Refusal rate was zero.

#### 1. Ecological performance of protected areas

We assessed ecological performance of protected areas by looking at the degree of human encroachment and the size and diversity of wildlife populations. Data on human encroachment were obtained from [6]. The 2010 Zambian census was used to obtain district-level estimates of human population growth rates [16].

Data on wildlife abundance in protected areas were derived from aerial census reports [17], [24], [25], [62]–[67]. The most recent reports were used, though some abundance estimates were made using census reports as old as 2003, and for some PAs no census data were available at all. Census data were available for 39 Zambian PAs (14 National Parks comprising 61,812 km^2^ and 25 GMAs comprising 152,122 km^2^) (or ∼93% of the national park and GMA estate). Estimates of mammalian biomass were made by removing species of bushbuck *Tragelaphus scriptus* size or smaller, hippopotamuses *Hippopotamus amphibius* and predators as most reports did not provide estimates for those species. The typical mass of an individual in a population for each species following [68] was multiplied by population sizes to estimate biomass.

We used rainfall, soil nutrient status and large herbivore biomass for 28 wildlife areas in eastern and southern Africa [69] to create five regression curves for predicting herbivore biomass for: 1) medium soil nutrient areas for moist-adapted species; 2) medium soil nutrient areas for arid-adapted species; 3) low soil nutrient areas for moist-adapted species; 4) low soil nutrient areas for arid-adapted species with annual rainfall <700 mm; 5) low soil nutrient areas for arid-adapted species with annual rainfall >700 mm. In each case, herbivore biomass was plotted against rainfall using the software programme GraphPad Prism. These five regression curves were then used to predict *potential* herbivore standing crop biomasses (kg/km^2^) for protected areas in which annual rainfall and soil nutrient data were available. Estimates for annual rainfall were determined from literature and internet sources, while soil nutrient status was determined using a combination of two sources: 1) soil maps [70] and 2) vegetation types identified from the literature and vegetation maps [71].

In protected areas where there was more than one soil or vegetation type, we estimated the proportion of each type within the area, and used these to calculate an average soil nutrient status. In many cases, the soil nutrient status estimated from soil and vegetation types corresponded well, but in cases when they differed, the lower estimate was selected for the sake of conservatism.

#### 2. Economic performance of protected areas

Data on earnings from photo-tourism in national parks, and from trophy and resident hunting in GMAs were obtained from ZAWA to assess economic performance of protected areas. Earnings of safari operators in GMAs were estimated using trophy off-takes for 2012 obtained from ZAWA, following [28] and using the mean 2013 pricing for Zambian trophy hunts (from a survey of *n* = 10 websites). Current per km^2^ earnings from trophy hunting in Zambia were compared with regional estimates derived from [28]. Further insights into the performance of the hunting and tourism industries were obtained from the literature.

#### 3. Social performance

Insights into the social performance of protected areas were obtained from the literature and through surveys. Comprehensive community surveys in GMAs have been completed by other authors recently (e.g. [8], [9], [30] and their findings provided insights into community-related issues.

### Ethics Statement

The University of Pretoria Ethics Committee approved this research, and approved the procedure for obtaining consent for the surveys conducted during the research. We were issued with written consent for this study from the Wildlife Producers’ Association of Zambia. In addition Zambia Wildlife Authority provided verbal approval and participated in the research. From respondents we obtained verbal consent prior to conducting the surveys. Written consent from individual respondents was not considered practical or necessary and the requirement for written consent was waived by the University of Pretoria. We documented any cases where respondents did not wish to participate in order to calculate refusal rates.

## Supporting Information

Figure S1 **The (a) biomass and (b) diversity of wild ungulates (excluding species of bushbuck size and smaller and hippos, for which data were unavailable for state protected areas) in GMAs, national parks and extensive (unfenced) private game ranches** [19] **.**
(TIFF)

Figure S2 **The (a) biomass and (b) diversity of wild ungulates (excluding species of bushbuck size and smaller and hippos, for which data were unavailable for state protected areas) in protected areas and extensive game ranches with and without NGO or private investment in law enforcement.**
(TIFF)
