# Supplementary figures and images for: Underperformance of African Protected Area Networks and the Case for New Conservation Models: Insights from Zambia
Source: PLoS One. 2014 May 21;9(5):e94109. doi: 10.1371/journal.pone.0094109 (PMC4029602; doi:10.1371/journal.pone.0094109)

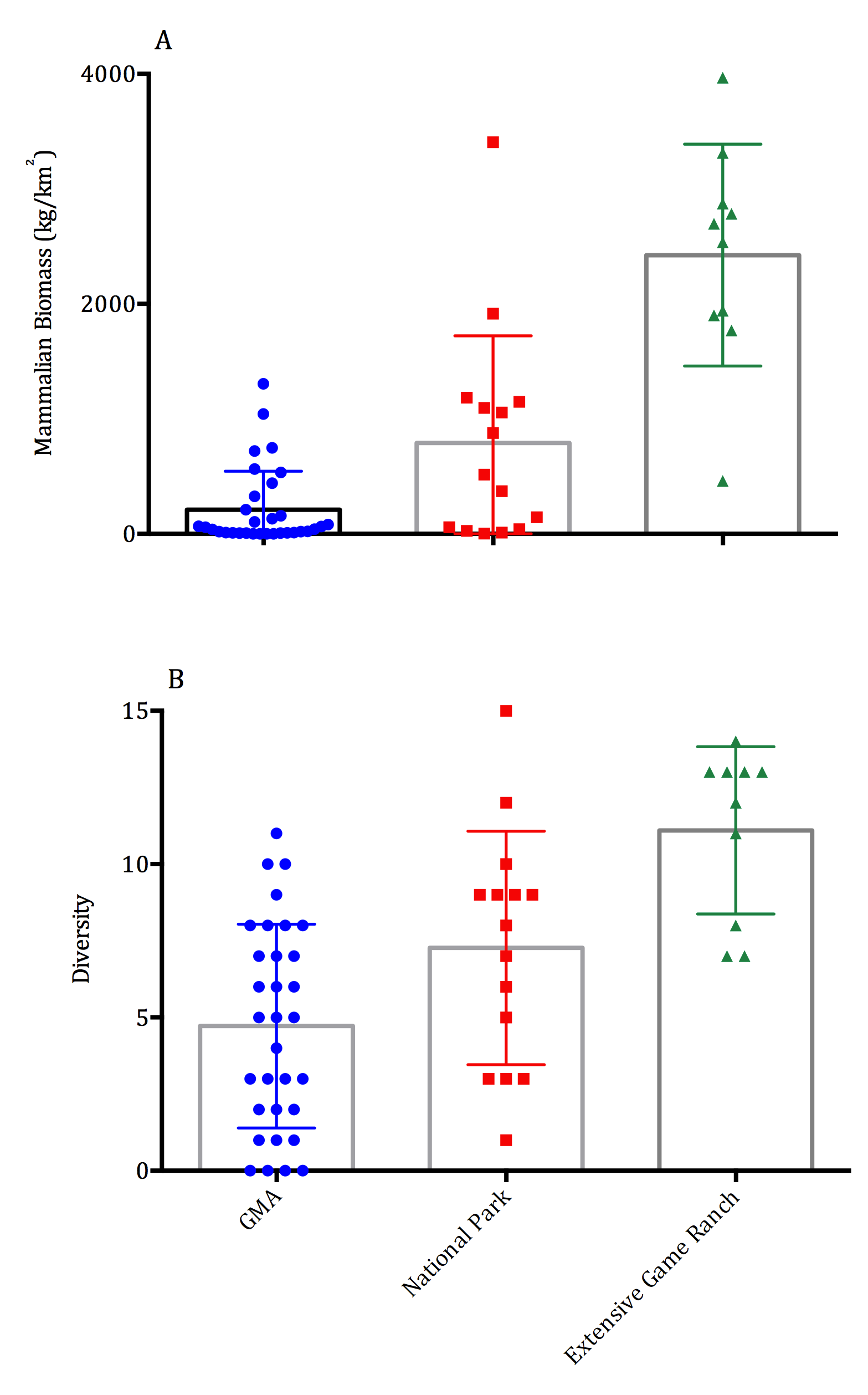

Supplement: Figure S1 — The (a) biomass and (b) diversity of wild ungulates (excluding species of bushbuck size and smaller and hippos, for which data were unavailable for state protected areas) in GMAs, national parks and extensive (unfenced) private game ranches [19] . (TIFF) [file pone.0094109.s001.tiff]

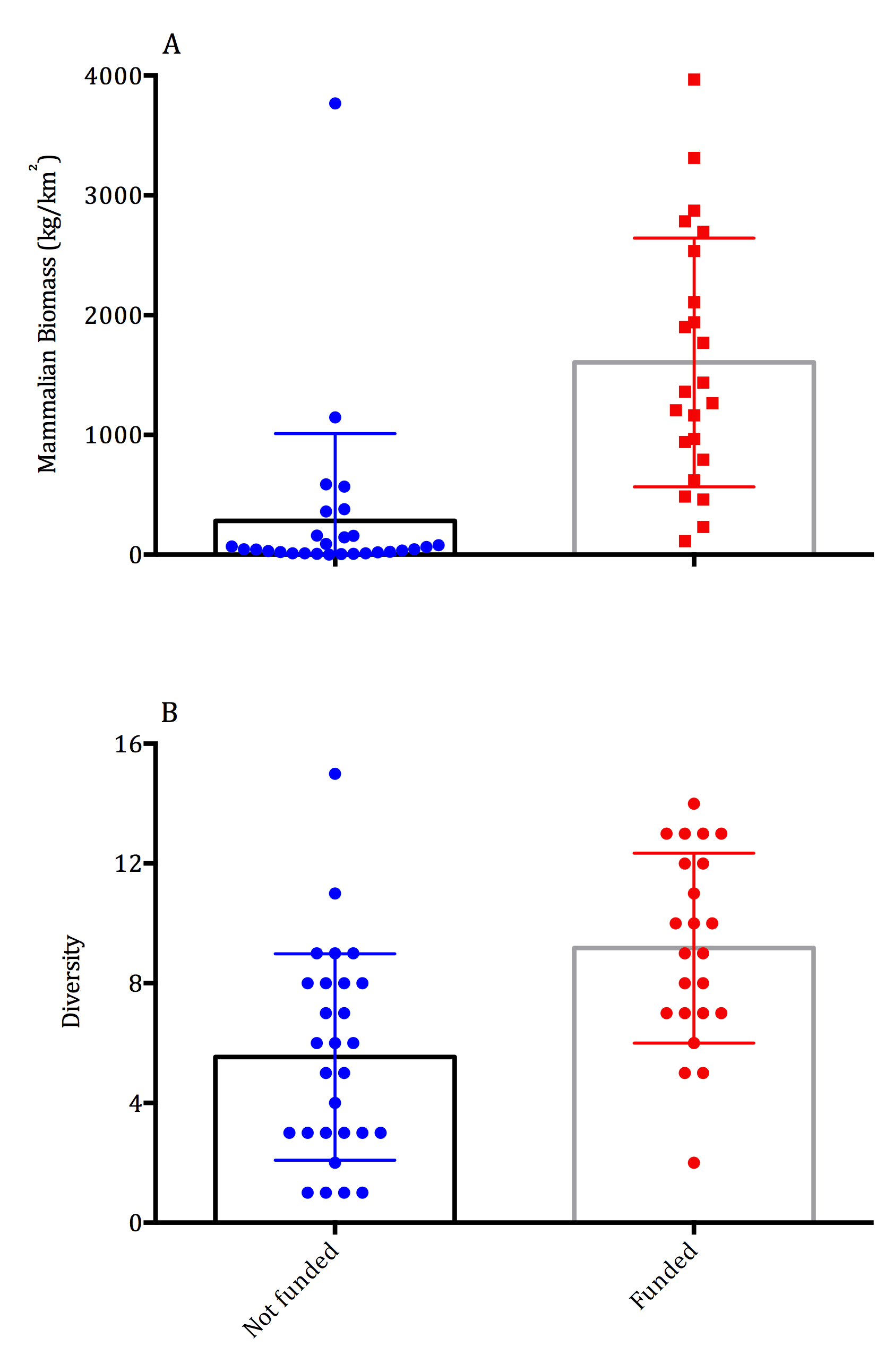

Supplement: Figure S2 — The (a) biomass and (b) diversity of wild ungulates (excluding species of bushbuck size and smaller and hippos, for which data were unavailable for state protected areas) in protected areas and extensive game ranches with and without NGO or private investment in law enforcement. (TIFF) [file pone.0094109.s002.tiff]
